# The role of BRCA1 in homologous recombination repair in response to replication stress: significance in tumorigenesis and cancer therapy

**DOI:** 10.1186/2045-3701-3-11

**Published:** 2013-02-06

**Authors:** Junran Zhang

**Affiliations:** 1 Department of Radiation Oncology, School of Medicine, Case Western Reserve University, 10900 Euclid Avenue, BRB 323, Cleveland, OH, 44106, USA

**Keywords:** BRCA1, Homologous recombination, Replication arrest, Poly(ADP-ribose) polymerase (PARP) inhibitor, DNA double strand breaks, Sister chromatid exchange

## Abstract

Germ line mutations in breast cancer gene 1 (BRCA1) predispose women to breast and ovarian cancers. Although BRCA1 is involved in many important biological processes, the function of BRCA1 in homologous recombination (HR) mediated repair is considered one of the major mechanisms contributing to its tumor suppression activity, and the cause of hypersensitivity to poly(ADP-ribose) polymerase (PARP) inhibitors when BRCA1 is defective. Mounting evidence suggests that the mechanism of repairing DNA double strand breaks (DSBs) by HR is different than the mechanism operating when DNA replication is blocked. Although BRCA1 has been recognized as a central component in HR, the precise role of BRCA1 in HR, particularly under replication stress, has remained largely unknown. Given the fact that DNA lesions caused by replication blockages are the primary substrates for HR in mitotic cells, functional analysis of BRCA1 in HR repair in the context of replication stress should benefit our understanding of the molecular mechanisms underlying tumorigenesis associated with BRCA1 deficiencies, as well as the development of therapeutic approaches for cancer patients carrying BRCA1 mutations or reduced BRCA1 expression. This review focuses on the current advances in this setting and also discusses the significance in tumorigenesis and cancer therapy.

## Two ended-DSB repair by HR: gene conversion (GC) versus crossover

DNA lesions result from errors in normal DNA replication, production of reactive oxygen species, and exposure to ultraviolet rays and ionizing radiation (IR). The damage induced by endogenous or exogenous insults can be base damages, single strand breaks (SSBs), DSBs, and intrastrand or interstrand cross-links [1]. The inability to properly repair damaged DNA can lead to genomic instability, a hallmark of cancer. To avoid the consequence of unrepaired DNA damage, multiple types of DNA repair mechanisms exist in cells to repair the various types of DNA damage on a regular basis, including base excision repair (BER), nucleic acid excision repair (NER), HR, single strand annealing (SSA), mismatch repair (MMR) and non-homologous end joining (NHEJ) [1]. HR is required for repairing multiple types of DNA damage including single stranded DNA (ssDNA), DSBs and DNA cross-links. In addition, HR is a critical mechanism for recovery of stalled or broken DNA replication forks. Certain genetic alterations, such as BRCA1 and BRCA2 mutations, are associated with increased risk of malignancy and enhanced sensitivity to chemotherapeutic agents, including PARP inhibitors. This review focuses on mechanistic aspects of the function of BRCA1 in HR repair following replication stress, and also the implications to tumor development and cancer therapy.

**Figure 1:**
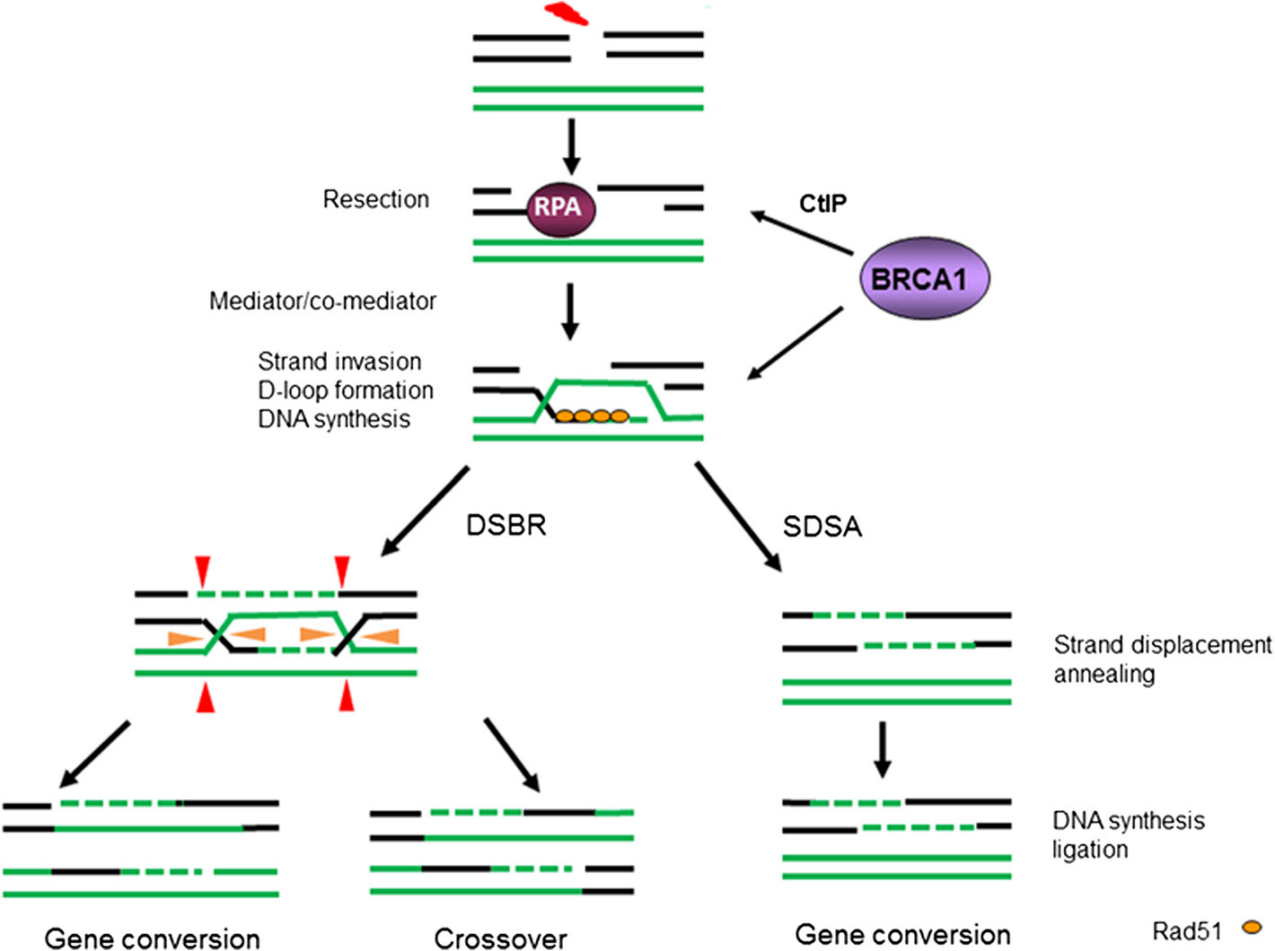
**DSBs can be repaired by several HR repair pathways including DSBR (double-strand break repair) and SDSA (synthesis-dependent strand annealing).** HR is initiated by resection of a DSB to provide 3’ ssDNA overhangs. Strand invasion by these 3’ ssDNA overhangs into a homologous sequence is followed by DNA synthesis at the invading end. After strand invasion and synthesis, the second DSB end can be captured to form an intermediate with two HJs. After gap-repair DNA synthesis and ligation, the structure is resolved at the HJs in a non-crossover (red arrow heads at both HJs) or crossover mode (orange arrow heads at one HJ and red arrow heads at the other HJ). Alternatively, the reaction can proceed to SDSA by strand displacement, annealing of the extended single-strand end to the ssDNA on the other break end, followed by gap-filling DNA synthesis and ligation. The repair product from SDSA is always non-crossover.

HR mediated repair can be triggered by DNA DSBs and blockages of DNA replication. The process of repairing DNA DSBs by HR has been extensively studied in both lower and higher organisms. In general, recombination is initiated at DSBs with the nucleolytic degradation of DNA ends to generate 3^′^-end ssDNA. This reaction is carried out by the MRE11/RAD50/NBS1 (MRN) complex [2,3]. CtIP plays a critical regulatory role in ssDNA resection, along with the MRN complex [4]. Once ssDNA is generated, it is rapidly bound by the ssDNA-binding-protein RPA, a protein consisting of three subunits: RPA1, RPA2 and RPA3. Through the action of recombination mediator/comediator proteins, RPA coated ssDNA is displaced by the RAD51 protein, a human homologue of *E. coli* RecA. The formed RAD51 nucleoprotein filament facilitates DNA strand invasion and exchange steps [5] which leads to formation of a Holliday junction (HJ) (Figure 1). From this point, the DSBR (double-strand break repair) pathway and the SDSA (synthesis-dependent strand annealing) pathway are defined. They are two primary models for how HR repairs two ended DSBs [6]. In the DSBR pathway, the second 3^′^ overhang also forms an HJ with the homologous chromosome, which most frequently is a sister chromatid. Whether recombination in the DSBR pathway results in crossover is determined by how the double HJs are resolved by a restriction endonuclease, a resolvase [7], which cuts only one DNA strand. RAD51C is an identified resolvase in mammalian cells [8]. Crossover occurs if one HJ is cut on the crossing strand and the other HJ is cut on the non-crossing strand (Figure 1). Alternatively, if the two HJs are cut on the crossing strands, gene conversion (GC) occurs without a crossover [9]. The DSBR pathway more frequently results in a crossover than GC (Figure 1). In the SDSA pathway, only GC occurs because the first invading 3^′^ strand is extended along the recipient DNA duplex by a DNA polymerase, and is released as the HJ resolves via branch migration. 

## DNA replication-associated lesions are repaired by HR via crossover

**Figure 2:**
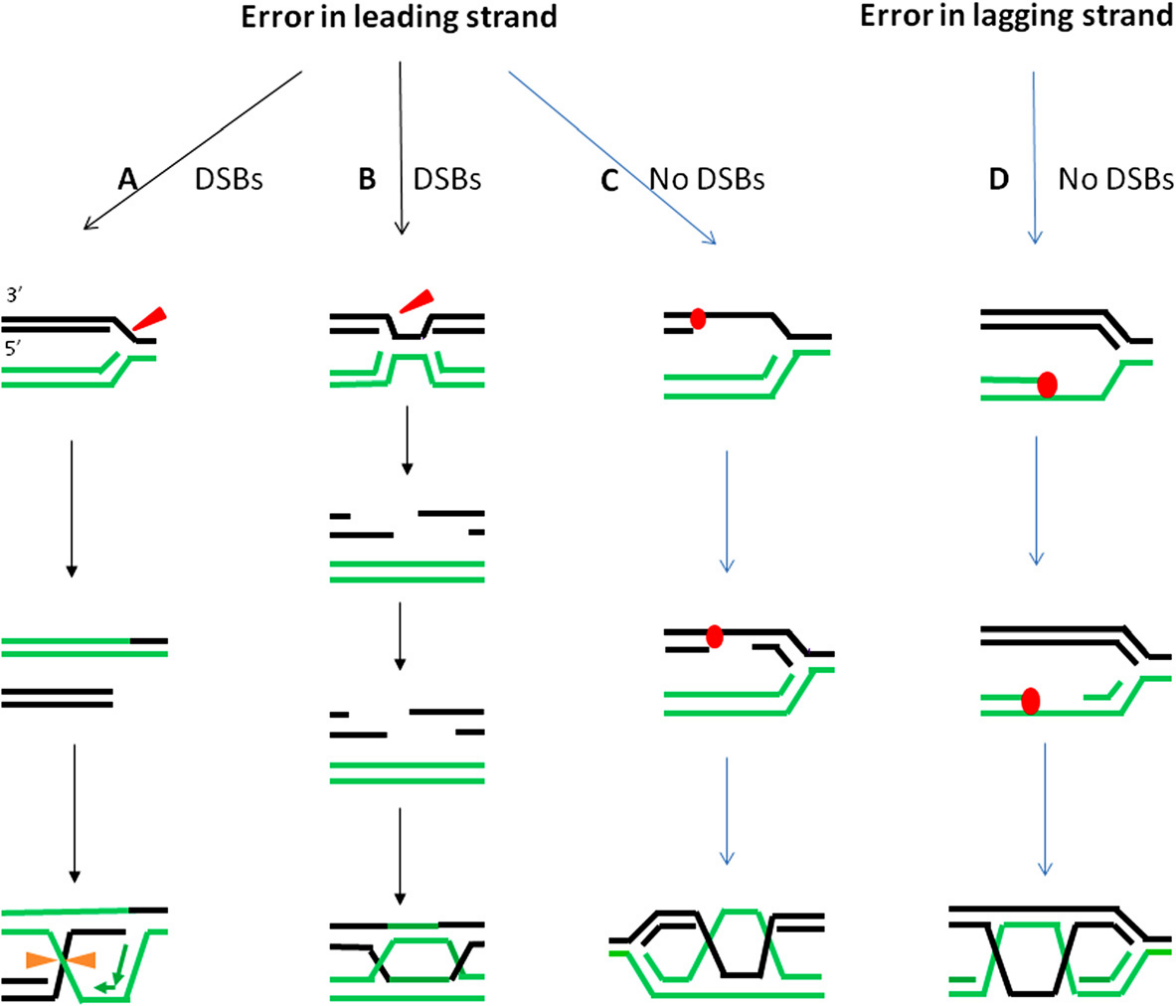
**Pathways of HR repair at stalled/ collapsed replication forks.** (**A,B,C**) Possible pathways resolving leading-strand blockages by HR. Stalled replication forks can be cleaved by an endonuclease to generate a one-sided DSB (A) which can be repaired by HR and re-establishment of a functional fork. Resolution of the single HJ in the orientation shown by the orange arrows results in SCE. Alternatively, a one sided DSBs can be converted into two sided DSBs by encountering a second replication fork; subsequently two end DSBs trigger HR by formation of double HJs (**B**). Moreover, uncoupling of lagging-strand synthesis can lead to downstream re-initiation of leading strand synthesis, resulting in a leading strand gap, which can be repaired by HR. In this situation, no DSBs are created (**C**). (**D**) Possible pathway resolving lagging strand blockage. Downstream re-initiation of lagging-strand synthesis after blockage leaves a gap on the lagging strand which can be repaired by HR.

The lesions occurring at stalled/collapsed replication forks can be repaired by HR or bypassed by translesion DNA synthesis (TLS). The HR mechanism required for repairing lesions at stalled or collapsed DNA replication forks in mammalian cells is less well-understood compared to the pathways identified in bacteria and yeast. There are several models available depending on whether the lesion occurs in the leading or lagging strands. If the lesion occurs in leading strands, stalled replication forks can be cleaved by an endonuclease, leading to the creation of a one-sided DSB. Similar to the RuvABC complex in *E. coli*[10]*,* the endonuclease Mus81 facilitates one ended DSB generation in mammalian cells [11,12]. One-sided DSB repair by recombination involves DNA strand invasion and one HJ formation (Figure 2A). A crossover is generated when the HJ structure is resolved [13]. Alternatively, a one-ended DNA DSB could subsequently progress to a two-ended DSB due to the firing of a new origin of replication under conditions of replication stress, and HR will be initiated to repair a structure that is similar to the classical two-end DSB (Figure 2B) [14]. In both situations (Figure 2A,B), DSBs are involved. In contrast, no DSBs are generated if uncoupling of continued lagging-strand synthesis with stopped leading strand synthesis occurs. Downstream re-priming of leading strand synthesis will result in the leading strand gap, and can be subsequently be repaired by recombination [10,15,16] (Figure 2C). If a lesion leads to lagging strand blockage (Figure 2D) the replication fork may not collapse. Downstream re-priming of lagging-strand synthesis after blockage leaves a gap on the lagging strand, which can be repaired by recombination [10]. Although it was reported that creation of DNA DSBs leading to replication fork collapse is a major mechanism to initiate HR in mammalian cells [14,17], it has been demonstrated that thymidine can potently induce HR in the absence of DSBs even after long term exposure [18]. Recent work from our lab showed that sister chromatid exchange (SCE, see discussion below) is induced when the cells are treated with 2 mM hydroxyurea (HU) for 6 hr , a condition in which no DSBs are detected by Comet assay or FISH [19]. Similar to lower organisms, therefore, HR can be induced in the absence of free DNA DSB ends in mammalian cells. 

Broken replication fork-stimulated HR may be different than HR induced by classical two-end DNA DSBs. Two-end DNA DSBs created by site-specific I-*Sce*I endonuclease overexpression in mammalian cells triggers HR repair by short gene conversion [20], whereas spontaneous HR, most likely occurring at replication forks, triggers repair via a SCE [21,22]. In addition, the product of HR induced by replication stress is SCE/long tract gene conversion [21,22]. Moreover, we have found that phosphorylation of RPA2 is specifically required for HR in response to replication arrest, but is not essential for the cutting two-end DSBs induced HR [23]. Further, GC detected by an I-*Sce*I based HR reporter is promoted by ATR, while SCE induced by replication fork collapse is suppressed by ATR [19]. These studies suggest that HR pathways required for repairing direct DSBs and replication blockage are distinct. Furthermore, HR pathways required for repairing replication fork stalling and collapse appear to be different as well. We have found that SCE induced by DSBs following fork collapse is suppressed by ATR, whereas the frequency of SCE induced by replication fork stalling is similar in cells with or without ATR depletion [19]. The complexity of the HR pathway was further increased by the observation that mouse cells lacking DNA POLβ, the major gap-filling DNA polymerase, display higher than normal SCE levels following alkylating agent exposure, although they exhibit normal levels of spontaneous SCE [13]. Also some HR proteins required for replication stress-induced SCE have no obvious effect in spontaneous SCE (see discussion below). In summary, HR repair pathways triggered during DNA replication blockage are differently regulated than those induced by direct DNA DSBs. In addition, several subtypes of the HR pathway exist to deal with spontaneous and induced DNA lesions resulting from replication fork stalling or collapse.

## SCEs are associated with DNA replication and HR

HR requires a template with sufficient sequence identity to the damaged strand in order to direct repair. In mammalian cells, the sister chromatid is the primary template for HR compared to the homologous chromosome [20]. SCEs occur naturally as events associated with normal DNA replication and upon replication fork stalling/collapse. Formation of SCEs is intimately associated with DNA replication because eukaryotic cells exposed to DNA-damaging agents in G_2_ show elevated SCE levels only after completing a subsequent replication cycle [24].

Although the molecular mechanisms controlling SCE are not fully understood, HR between sister chromatids is principally responsible for SCE in higher eukaryotic cells [25]. This process is considered to be conservative and error-free, since no information is generally altered during reciprocal interchange by HR. It is known that not all types of DNA damage give rise to SCE. DNA DSB agents can not efficiently induce SCEs. In contrast, SCEs can be induced by various genotoxic treatments causing replication arrest. S phase-dependent agents, such as mitomycin C (MMC) and UV light are among the most effective inducers of SCE [26], presumably the conditions that increase the cellular burden of SSBs or subsequent DSBs creation during replication stress generally induce SCE efficiently. Thus, the simplest pathway by which SCE likely occurs is through HR-mediated restart of a broken DNA replication fork when it encounters a nick or gap in one parental strand [13] (Figure 2A).

Many HR proteins have been reported to promote SCE in chicken DT40 cells. HR defective mutants, including mutants of RAD51, RAD54, and the RAD51 paralogs (i.e. RAD51B, C, and D and XRCC2), consistently have reduced SCE [25][27]. However, in mammalian cells, the results are more complex. Rad54 knockout mice cells show little or no reduction in spontaneous SCE, but there is a noticeable deficiency in MMC-induced SCE [28,29]. Moreover, some RAD51 paralog mutants show modest reductions in SCE, but isogenic *rad51d* mutant lines in both chinese hamster ovary and mouse fibroblasts show no decrease in spontaneous SCE [29,30]. Consistent with these studies, we observed that BRCA1 has no obvious role in spontaneous SCE (unpublished data), although BRCA1 promotes replication-stress induced SCE. Although HR is considered to be the pathway for formation of SCEs, the observation that in HR-deficient cells, the background SCE levels are comparable to the parental cells suggests that spontaneous SCEs do not originate from HR. On the contrary, HR seems to be involved in the formation of induced SCEs [31]. In summary, the variation in phenotypes between spontaneous and induced SCE suggests that more than one molecular pathway is responsible for SCE in response to replication stress.

In contrast to HR proteins, several proteins were found to suppress SCE. The helicase protein, BLM, appears to be important in this process since loss of the *BLM* gives rise to an elevated frequency of SCE during DNA replication [32]. BLM suppresses SCE via multiple processes, including through association with topoisomerase IIIα (hTOPO IIIα) [33-35] and/or RAD51 [36][37]. It has been suggested BLM and hTOPO IIIα together effect the resolution of a recombination intermediate containing a double Holliday junction[38]. Although it is believed that BLM works as an anti-recombinase, in *Drosophila* DmBlm was found to be required specifically to promote the SDSA, a type of HR associated with GC but not cross-over (Figure 1). This result was confirmed in the chicken DT40 B lymphocyte line by demonstrating that Ig GC frequency was drastically reduced in *BLM*^−/−^ cells [39]. Thus, BLM suppresses SCE but promotes GC.

Recent work in our lab showed that ATR suppresses SCE upon replication fork collapse, although ATR has no role in SCE when the replication forks stall [19]. HU, which functions as an inhibitor of ribonucleotide reductase, slows down fork progression by reducing dNTP pools, leading to stalled replication forks that after prolonged treatment collapse into DNA DSBs [14]. We found that ATR depletion leads to an increased rate of SCE in the cells treated with HU for 18 hr when DSBs are efficiently created. Conversely, ATR depletion suppressed I-SceI-induced GC [19]. Although it is not clear how ATR suppresses SCE, there are several possibilities. First, the similar effect of ATR and BLM deficiency on SCE and GC suggest that both proteins act in the same pathway, presumably ATR suppresses SCE via regulation of BLM. BLM is phosphorylated by ATR on two residues, Thr99 and Thr122, and has a role in the recovery from S-phase (16) [40]. Surprisingly expression of BLM containing T99A and T122A substitutions in human BLM defective cells was able to suppress the hyper-SCE phenotype, which is the same as expression of wild type BLM, indicating that substitution of Thr99 and Thr122 with alanine did not prevent BLM from suppressing spontaneous SCE [40]. Thus, BLM phosphorylation by ATR has no direct role in spontaneous SCE. However, the possibility that BLM phosphorylation by ATR is important to SCE induced by replication stress has not been tested. Alternatively, the SCE repression by ATR may operate in part by impeding the resection of cutting free DNA ends. It has been reported that the MEC1 replication checkpoint suppresses the formation of RAD52 foci and prevents HR at chromosome breaks induced by the HO endonuclease in yeast [41]. This repression operates at least in part by impeding resection of DNA ends, which is essential to generate the 3^′^ ssDNA tails that are the primary substrate of HR. Interestingly, the MEC1 pathway does not prevent recombination at stalled forks, presumably because they already contain ssDNA [41], which is consistent with that the concept that ATR has no role on SCE following replication fork stalling but suppresses SCE following fork collapse after DSBs are produced [19]. Lastly, the elevated SCE frequency following ATR depletion may be related to the specific locations where the increased breakages occur. Chromosomal fragile sites are the regions of the genome which exhibit gaps or breaks on metaphase chromosomes under conditions of partial replication stress [42]. Common fragile sites with or without associated breakages are the preferred location for SCE in aphidicolin treated cultures [43,44]. SCEs were found to be distributed nonrandomly across fragile sites and nonfragile sites; and among the fragile sites, the high frequency SCE sites were highly correlated with the high frequency breakage sites [44], indicating that SCE are preferentially induced at common fragile sites with broken ends. ATR protein was found to bind to three regions of FRA3B under conditions of replication stress, and a deficiency of ATR results in a dramatic increase in fragile site breakage [45,46]. Thus, defective ATR signaling could result in DNA breakages at the sites which are the hotspots for SCE.

## The role of BRCA1 in HR

Germ line mutations in BRCA1 confer increased susceptibility of developing breast cancer with high penetrance [47,48]. BRCA1 function may also be lost in a substantial number of sporadic breast cancers [49-52]. The BRCA1 protein contains multiple functional domains, including a highly conserved N-terminal RING finger which contributes to its E3 ligase activity. BRCA1 interacts directly or indirectly with numerous molecules [53], which is consistent with the observation that BRCA1 deficiency results in pleiotropic phenotypes, including defective DNA damage repair, defective cell cycle checkpoints, increased apoptosis, impaired spindle checkpoint and chromosome damage [54,55]. Although it is not clear if all observed phenotypes contribute to BRCA1 mutations associated tumorigenesis, the function of BRCA1 in HR repair plays a critical role in BRCA1 associated cancer development [55,56].

The observation that BRCA1 associates and colocalizes with RAD51 in nuclear foci in mitotic cells is one of the earliest indications that BRCA1 functions in HR repair [57]. These foci have been observed before and after DNA damage [58-60], indicating the role of BRCA1 in repair of intrinsic or induced DNA damage. Further evidence comes from the observation that BRCA1-deficient cells are highly sensitive to IR and display chromosomal instability including chromatid breaks, a chromosome abbreviation frequently observed in cells with HR deficiency [61,62]. There is direct evidence that BRCA1 plays a role in HR. Two reports found that BRCA1 deficiency in mouse embryonic stem cells leads to decreased HR repair of direct DSBs induced by the site-specific I-*Sce*I endonuclease [63,64]. Impaired HR in human cancer cells depleted of BRCA1 using a similar HR reporter has also been observed [65]. In addition there is a report implicating BRCA1 in *Ig* GC [66]. While the focus of BRCA1 investigation has been on DSB processing, its potential role in dealing with replication stress is relatively less explored. The observation that BRCA1 is required for subnuclear assembly of RAD51 and survival following treatment with a DNA damaging agent that does not cause DNA DSBs suggests that BRCA1 is involved in HR upon replication fork stalling. Our recent work has suggested that both BRCA1 and RAD51 proteins are co-localized with ssDNA regions following HU treatment for 6 hr when no DSBs is detected. In addition, a reduced proportion of cells with RAD51 foci and SCE frequency were observed in the cells with BRCA1 depletion under the same conditions. This observation suggests a role of BRCA1 in HR via regulation of RAD51 recruitment in the absence of DNA DSBs [19]. This study was the first to shed light on how BRCA1 deficiency influences HR repair in the context of a stalled replication fork. In addition, a recent report by Pathania et al. demonstrated that BRCA1 is important in dealing with UV-induced DNA lesions without detectable DNA DSBs [67]. In the model proposed by the authors, the UV sensitivity of BRCA1-deficient cells may be a compound phenotype from the perturbed intra-S phase and G2/M checkpoints, lesion removal, and TLS. However, given the fact that SCE can be induced by UV light [68] and HR contributes to cell viability after UV-light treatment [69], the role of BRCA1 in SCE at stalled replication forks would be an alternative mechanism contributing to the observed UV sensitivity in BRCA1-deficient cells. In addition to the role in SCE at stalled replication forks, BRCA1 is important for SCE produced by fork collapse as well. However, the role of BRCA1 in replication fork collapse-induced SCE is suppressed by ATR whereas the role of BRCA1 in promoting SCE following replication fork stalling is intact with or without ATR depletion. Thus, we conclude that BRCA1 facilitates SCE via distinct mechanisms when replication forks stall or collapse. Interestingly, it has been previously demonstrated that BRCA2-defective hamster cells are impaired in short tract GC but maintain proficiency in SCE [70]. Thus, it would be very interesting to test how BRCA2 regulates SCE when ATR is defective.

The question of why BRCA1 plays a profound role in replication fork collapse-induced SCE in cells with ATR deficiency remains open. One possibility is that SCE rate is very low in the normal context because inappropriate template choice within a sister chromatid leads to unequal SCE leading to gene duplication or deletions, which are associated with tumorigenesis [71]. However, SCE becomes a major mechanism to repair DNA DSBs when cells with a defective checkpoint signaling are challenged by replication stress. Therefore, the role of BRCA1 in SCE-associated HR is not evident unless ATR is depleted. Alternatively, loss of ATR may provide ideal substrates for BRCA1 in HR. BRCA1 promotes repair of DSBs following replication fork collapse via multiple mechanisms whereas BRCA1 promotes HR following replication fork stalling solely via the facilitation of ssDNA resection (see discussion below). Last, ATR may play a direct role in BRCA1-dependent SCE following replication fork collapse via phosphorylation of BRCA1. However, it is unclear how ATR-dependent phosphorylation of BRCA1 alters BRCA1 activities in SCE when replication arrests.

Of note, recombination related processes have a central function in the recovery of stalled or collapsed replication forks in both bacteria and eukaryotic cells [10,72]. For example, the endonuclease Mus81 in mammalian cells contributes to replication restart by promoting HR via facilitation of one-ended DSBs generation [12]. However, it was reported recently that HR facilitates repair of DSBs following fork collapse but does not necessarily contribute to replication fork restart in mammalian cells [14]. It was found that stalled replication forks are efficiently restarted in a RAD51-dependent process that does not trigger HR. In contrast, replication fork collapsed by prolonged replication blocks do not restart, and global replication is rescued by new origin firing. Thus, it would be very interesting to evaluate whether the role of BRCA1 in SCE contributes to recovery of stalled/collapsed replication forks.

## BRCA1 function in HR: a recombination mediator/comediator and promoting ssDNA resection

The interaction of both BRCA1 and BRCA2 with RAD51 suggests a functional link between the three proteins in the RAD51-mediated DNA damage repair process. However, while BRCA2 is directly involved in RAD51-mediated repair, BRCA1 seems to act in a more complicated mechanism via an interaction with other proteins [73,74] (Figure 1). Although the mechanisms by which BRCA1 functions in HR have not been clear, studies have suggested that BRCA1 acts as a recombination mediator/comediator, and promotes ssDNA resection via interaction with CtBP-interacting protein (CtIP). Recombination mediators are proteins facilitating displacement of RPA by RAD51 by binding RAD51 and possess a high affinity for ssDNA [5]. The mediators help overcome the suppressive effect of RPA by targeting RAD51 to free DNA or DNA already covered with RPA. To assist these recombination mediators, a second group of proteins is required and we define these proteins here as ‘recombination co-mediators’ [75]. BRCA2 is a well-defined mediator by direct interaction with RAD51 protein. RAD52 may function as an HR mediator when BRCA2 is absent in mammalian cells [76]. In general, in the absence of mediator/comediator, RAD51 overexpression can partially compensate. BRCA1 might act as a mediator/comediator since overexpression of RAD51 in BRCA1-deficient DT40 cells rescues defects in proliferation, DNA damage survival, and HR support [75,77]. A second molecular mechanism by which BRCA1 acts in HR is via association with CtIP [78]. The work from Yun et al. suggested that the function of CtIP in HR induced by I-*SceI* overexpression is dependent on BRCA1 recruitment and the phosphorylation of S327, which mediates its interaction with BRCA1 [79,80]. Cells expressing CtIP protein that cannot be phosphorylated at S327 are specifically defective in HR and have a decreased level of ssDNA induced by X-rays. The report supports a model in which phosphorylation of CtIP S327 as cells enter S phase, and the recruitment of BRCA1, functions as a molecular switch to shift the balance of DSB repair from error-prone DNA end- joining to error-free HR via facilitating ssDNA resection [78]. The possibility that BRCA1 functions in ssDNA resection via association with CtIP during DNA replication arrest came from our recent publication [19]. We found that CtIP depletion leads to a similar pattern in SCE formation when replication fork arrest compared to those occurring in cells with BRCA1 depletion, namely CtIP knockdown leads to decreased frequency of SCE following replication fork stalling independent of ATR. In contrast, the CtIP knockdown leads to an obviously decreased SCE frequency in cells depleted of ATR after 18 hr HU treatment when obvious DSBs are generated, although it has only a minor effect on SCE formation in cells with intact ATR expression. This result is similar to that observed in cells with BRCA1 knockdown, indicating that CtIP may function in the same pathway as BRCA1 [19].

The questions of whether the molecular mechanism by which BRCA1 promotes SCE after replication fork stalling or collapse is similar has not been resolved. However, it appears that BRCA1 may function differentially [19]. We found that BRCA1 depletion leads to a decreased RPA2-phosphorylation by immunoblotting in cells following 6 hr of HU treatment in the absence of detectable DNA DSBs. Conversely, BRCA1 depletion had no obvious effect on RPA2-phosphorylation in cells following 18 hr of continuous HU treatment. The likely scenario is that loss of BRCA1 leads to a defect in ssDNA resection when replication forks stall, which results in the impaired RPA2-phosphorylation. However, when replication forks collapse, BRCA1 also functions as a mediator of RAD51, and the loss of BRCA1 should lead to increased RPA2-phosphorylation due to a defective RAD51 recruitment, counteracting the decreased RPA2 phosphorylation resulting from impaired ssDNA resection. Thus, the levels of RPA2 phosphorylation are similar in cells with or without BRCA1 depletion when replication forks collapse. The idea that ssDNA resection occurs during replication stalling is supported by evidence from both bacteria and mammalian cells. In *E.coli*, ssDNA resection is required to enlarge the ssDNA gap for RAD51 dependent HR [81]. Studies in mammalian cells have shown the existence of ssDNA gaps during stalled DNA replication in UV-damaged S phase cells [82,83]. RPA-coated ssDNA regions upon UV damage were much reduced in the absence of BRCA1 [67], suggesting a role for BRCA1 in DNA resection when replication forks stall. A model for the role of BRCA1 in HR in response to replication fork stalling or collapse has been proposed in our recent publication [19].

The function of BRCA1 in ssDNA resection is regulated by 53BP1 and RPA80. The crosstalk between BRCA1 and 53BP1 in ssDNA resection has been highlighted in recent studies. These studies showed that 53BP1 inhibits HR in BRCA1-deficient cells via a blocking resection of DNA breaks [84-86]. Unlike Brca1 mutants, Brca1*/*53BP1 double mutants are proficient for HR, and assemble RPA foci after DNA damage, arguing that the primary function of BRCA1 in DSB repair is to promote resection by antagonizing 53BP1. These studies also found that loss of 53BP1 restores the deficiency of PARP inhibitor induced SCE in MEF cells with BRCA1 deficiency [84]. Thus, it would be very interesting to determine how 53BP1 affects the role of BRCA1 in SCE formation in response to replication fork stalling or collapse in the future. In addition, a recent study from Hu et al. suggests that RAP80 contributes to the suppression of exaggerated, BRCA1-dependent HR activity [87]. It was found in this study that the rate of SCE induced by etoposide, a potent DSB inducer, is higher in RAP80-depleted cells compared to that observed in control cells. Thus, RAP80/BRCA1 complexes suppress excessive DSB end processing. However, the available data cannot explain how 53BP1 and RAP80 function in the different settings. For instance, the regulation of 53BP1 in ssDNA resection occurs in BRCA1 deficient cells. In contrast, suppressing BRCA1-driven HR by RAP80 can occur in cells with intact BRCA1. Further studies are needed to address these questions.

A very recent publication suggests BRCA1-associated exclusion of 53BP1 from DNA damage sites from examining the spatial distribution of BRCA1 and 53BP1 proteins within single IR induced focus (IRIF) by employing a novel super-resolution microscopy: three dimensional structured illumination microscopy [88]. The authors found that as cells transition through S-phase the recruitment of BRCA1 into the core of IRIF, which they assume involves HR, is associated with an exclusion of 53BP1 to the focal periphery, leading to an overall reduction in 53BP1-chromatin occupancy. The same pattern was also observed after treatment with camptothecin, a Topoisomerase I inhibitor that induces DSBs in S-phase when replication forks encounter trapped Top1-DNA cleavage complexes. Therefore, the authors propose that BRCA1 antagonizes 53BP1-dependent DNA repair in S-phase by inhibiting its interaction with chromatin proximal to damage sites. How the molecular choreography of 53BP1, BRCA1 and other proteins take place and how this physical distribution in a focus affects the function of BRCA1 in HR, however, is not yet clear.

## Is BRCA1 E3 activity required for HR?

One of the functions of BRCA1 is as an E3 ligase. BRCA1 ubiquitin ligase activity is observed when BRCA1 forms a heterodimeric complex with BARD1 [89]. The potential importance of the E3 ligase activity of BRCA1 in cellular pathways is supported by the observation that missense mutations within RING finger domain of BRCA1, which cause familial breast cancer, abolish the E3 activity [89-93]. The role of BRCA1 E3 activity in HR has been reported in several publications. BRCA1 transgenes with E3 ligase mutations are unable to restore HR in BRCA1 defective cells using I-*SceI* based HR reporters detecting GC [91,94]. In addition, the investigation of multiple mutants of BRCA1 from patients that disrupt the interaction of E2 enzymes without perturbing the BRCA1–BARD1 complex has revealed that E3 ligase activity strongly correlates with BRCA1 functions in HR, and breast cancer susceptibility [77,94]. Interestingly, many studies have suggested a role for the E3 ligase activity of BRCA1 in HR in repairing two-ended DSBs. However, surprisingly, genetically engineered mouse ES cells expressing BRCA1 with a substitution of alanine for isoleucine at position 26 (I26A), a frequent mutation that disrupts the binding to the E2 subunit without perturbing BARD1 binding [90], do not exhibit HR failure [95]. The ES cells with BRCA1 I26A are resistant to genotoxic stress and are capable of accumulating RAD51 at DSBs, and mediate HR repair at the same level as cells with wild type BRCA1. This report questions the importance of E3 ligase activity of BRCA1 to HR. However, the same study also demonstrated that an E3 ligase mutation in BRCA1 leads to a decrease in recombination mediated gene targeting [95]. Since it has been shown that gene targeting occurs through a process in which only a one-ended DSB is involved [96], it is possible that the E3 ligase activity of BRCA1 is only required for HR induced by a one-ended DSB but is not important for HR induced by two-ended DSBs. So it is possible that the E3 ligase activity of BRCA1 is required for the HR process in response to some specific types of DNA damage, such as HR triggered when replication forks are blocked. This hypothesis is supported by the same study demonstrating that the ES cells expressing inactive BRCA1 E3 ligase show an elevated level of damage-induced, but not spontaneous, chromosomal abnormalities [95]. To clarify the mechanisms behind these observations, there is a need to study systematically how E3 ligase activity of BRCA1 regulates HR under replication stress conditions. Any advances in this topic would advance the current knowledge of BRCA1 associated breast cancer development.

## The role of BRCA1 in HR in response to replication stress and tumor prevention

A phenotypic hallmark of cells with mutations in genes involved in HR is chromosome instability. In the absence of HR, the resulting phenotypes can be seen either by spectral karyotyping (SKY) or by array-comparative genomic hybridization (aCGH), which detects large losses and gains across the genome that are common in BRCA1-deficient cells [73]. Genomic instability following loss-of-function of BRCA1 is hypothesized to be a key factor leading to tumorigenesis in individuals with BRCA1 mutations. It is generally believed that BRCA1 maintains genomic stability by promoting error free HR and suppresses error prone NHEJ [97-99]. This idea was further confirmed by a recent report demonstrating that knockdown or loss of the BRCA1 protein results in an increased frequency of plasmid DNA mutagenesis and microhomology mediated end joining following a DSB, suggesting that that BRCA1 protects DNA from mutagenesis during nonhomologous DSB repair [100].

Tumorigenesis due to loss of BRCA1 is a consequence of genetic instability. Numerical and structural aberrations were initially found by SKY analysis in murine embryos carrying a Brca1 null mutation [61]. Later, it was observed that mouse embryonic fibroblast (MEF) cells carrying a targeted deletion of exon 11 display extensive chromosomal abnormalities and a defective G_2_/M checkpoint [62]. Although the function of BRCA1 in HR was not discussed in the study, chromatid breaks and quadriradial chromosome, two types of featured chromosomal aberrations frequently observed in cells with HR deficiency, were observed in Brca1^Δ11/Δ11^ MEFs. The studies from a different group also suggested that spontaneous chromosomal instability, including chromatid breaks and exchanges and chromosome breaks, deletions, and translocations are significantly higher in *Brca1*^−/−^ cells as compared with Brca1^+/+^[64]. Moreover, dramatic chromosome aberrations were noted in cells deficient in Brca1 [101]. All of the data uncover an essential role of BRCA1 in maintaining genetic stability through numerous functions including HR. Nevertheless, chromosome abnormalities in cells without BRCA1 may not necessarily result from dysfunctions in HR. A recent work from Bunting et al. suggests that BRCA1 functions independently of HR in DNA interstrand crosslink repair [102]. The authors found that *Brca1*^*Δ11/Δ11*^ cells were hypersensitive to two intra- or interstrand crosslinking drugs, nitrogen mustard and MMC. 53BP1 depletion restored HR in Brca1 ^*Δ11/Δ11*^ cells but did not restore the sensitivity and chromosome aberrations including chromosome and chromatid breaks and radiation structures, indicating that BRCA1 has a function in crosslink repair and maintaining genomic stability during replication arrest which is separate from its role in HR.

## Loss of genes required for cell cycle checkpoints and BRCA1 associated tumorigenesis

Cells with damaged DNA frequently arrest, which reduces the probability of progressing to malignancy. Mutations in checkpoint pathways can permit the survival or continued growth of cells with genomic abnormalities, thus enhancing the likelihood of malignant transformation [103]. This is no exception for BRCA1 mutation associated tumor development. Loss of BRCA1 leads to embryonic lethality. Multiple studies suggest that the p53 loss cooperates with the loss of BRCA1 in tumorigenesis [61,104-109]. In addition, other genes required for cell cycle checkpoint, including ATM, CHK2 and ATR, seem to be important also for BRCA1-mutation tumorigenesis. Loss of Atm or Chk2 rescues the embryonic lethality of Brca1 mutant mice and leads to the development of multiple tumors [110]. In addition, ATM expression can be aberrantly reduced or lost in tumors expressing BRCA1 or BRCA2 mutants compared with sporadic tumors without BRCA1 or BRCA2 mutations [111]. Epidemiological evidence implicates that Chk2 and BRCA1 are in the same breast cancer prevention pathway [112], which is supported by the molecular process controlled by their interaction. Chk2 phosphorylates the serine 988 (S988) residue of BRCA1 and co-localizes with BRCA1 within discrete nuclear foci prior to DNA damage by γ-irradiation [113]. This phosphorylation is critical for the ability of BRCA1 to restore survival after DNA damage in BRCA1-mutated cell lines. In addition, the studies from our lab and others show that prevention of Chk2-mediated phosphorylation via mutation of the S988 of BRCA1 disrupts both HR detected by *I-SceI* reporter and the suppression of error prone-NHEJ [97-99], supporting the hypothesis that Chk2-dependent phosphorylation modulates the function of BRCA1 [97,99]. Moreover, uterus hyperplasia and increased carcinogen-induced tumorigenesis in mice carrying a targeted mutation of the Chk2 phosphorylation site in BRCA1 has been reported [114], suggesting that Chk2 phosphorylation is involved in the BRCA1 function in repressing tumor formation. An interesting question would be whether the role of BRCA1 in SCE induced by replication arrest is regulated by Chk2 phosphorylation.

ATR signaling regulates several cell cycle checkpoints and induces S-phase arrest in response to replication stresses [115]. Although there is no data available for the role of ATR in BRCA1 associated tumorigenesis in animal models, a linkage of ATR with BRCA1 was suggested by earlier cell biology and biochemistry studies. ATR can phosphorylate BRCA1 on several residues [116-118]. Moreover, ATR colocalizes with BRCA1 in foci in cells synchronized in S phase and after exposure to DNA damaging agents or DNA replication inhibitors, associating BRCA1 and ATR with the response to stalled replication forks [117,118]. Furthermore, the dramatic relocalization of ATR nuclear foci in response to DNA damage overlaps with the nuclear foci formed by BRCA1. In addition to cell biology and biochemistry studies, it has been reported that ATR was down-regulated in BRCA1 mutation carriers following radiation using high-density cDNA microarray technology [119]. In this study, the expression profiles of breast fibroblast samples from nine heterozygous BRCA1 mutant carrier individuals were compared to the profiles of five reduction mammoplasty fibroblast samples with a very low probability of the presence of BRCA1 mutations as controls. All of the samples were short-term primary cultures, and were irradiated to induce sublethal DNA damage. ATR was found to be down-regulated in mutation carriers compared with the controls, indicating a potential role of BRCA1 in ATR expression because of its decreased transcription [119], and further suggesting that ATR may be involved in BRCA1 associated tumorigenesis. Since the function of BRCA1 in SCE following replication fork collapse is more profound when ATR is depleted, this could be another molecular mechanism explaining why a second mutation in cell cycle checkpoint genes is important for BRCA1 associated cancer development in addition to permitting survival of the cells with BRCA1 mutations.

## The role of BRCA1 in HR following replication stress: implications in PARP-inhibitor therapy

**Figure 3:**
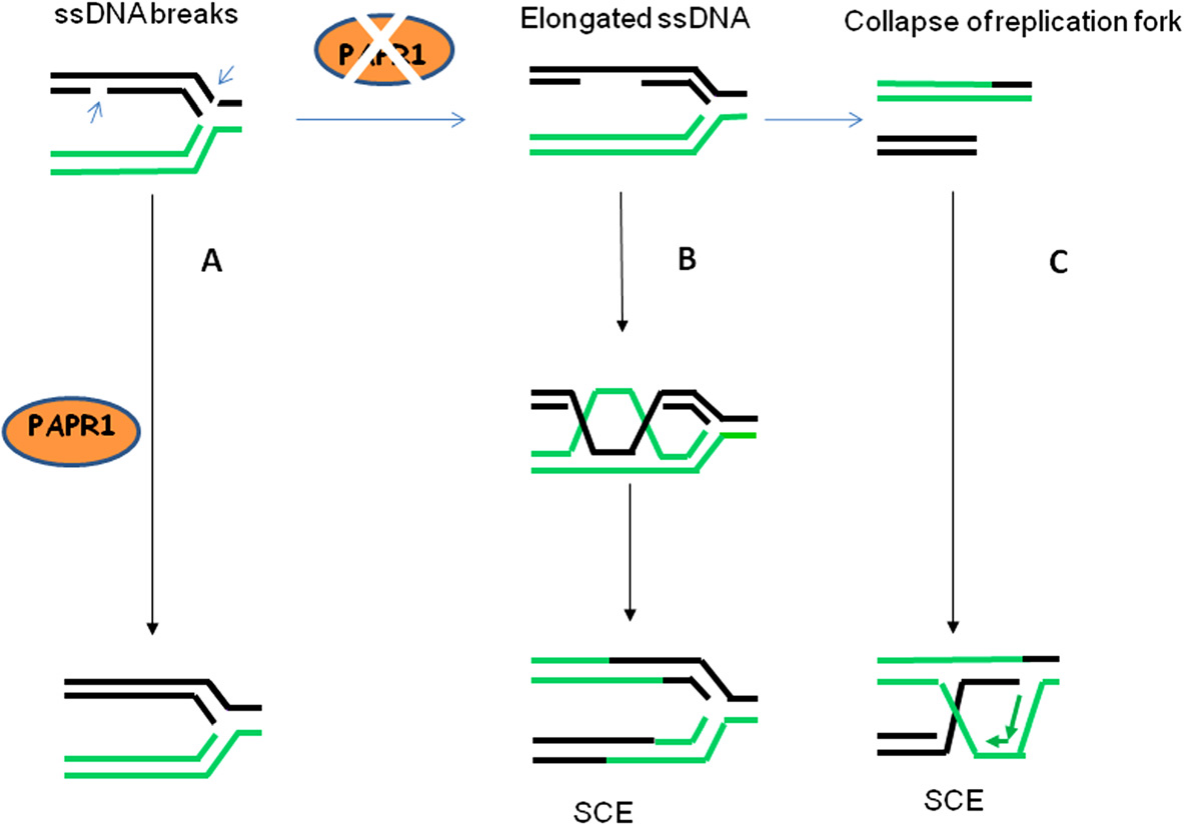
**DNA lesions caused by PARP inhibitors lead to increased crossovers.** DNA breaks are detected by PARP1 and PARP1 is active in response to DNA breaks. In the cells with intact PARP1 activity, the ssDNA is efficiently repaired (**A**). However, when the PARP1 activity is inhibited, unrepaired ssDNA breaks can be converted into elongated ssDNA (**B**) or subsequently into DSBs due to replication collapse (**C**). Both DNA structures stimulate SCE via HR.

PARP inhibitors have been shown to be selectively lethal to cells deficient in BRCA1 or BRCA2 due to synthetic lethality [120-123]. The PARP family consists of 17 proteins based on structural similarity. PARP1 is the protein that is best understood. This protein detects and binds to sites of ssDNA damage, and then synthesizes poly (ADP) ribose (pADPr, PAR) and transfers it to acceptor proteins. The acceptor proteins include PARP1 itself and other proteins involved in DNA repair, such as XRCC1, a protein involved in BER [124]. However, a recent report from Ström et al. suggested a distinct role of XRCC1 and PARP inhibition in SSB repair [125]. No direct role for PARP1 in BER was observed, but that PARP inhibitors trap PARP on the SSB intermediate formed during BER. Therefore, PARP1 plays an essential role in the latter steps of BER ligation. It is widely believed that the reason that recombination defective cells are sensitive to PARP inhibitors is because GC-associated HR has an important role in repairing a DSB. Thus, the increased number of unrepaired endogenous SSBs in PARP inhibited cells result in more collapsed replication forks, which require GC-mediated HR for repair [126]. In a BRCA1/2 defective background these DSBs are likely to be repaired by more error-prone repair mechanisms, causing chromosome aberrations and loss of viability [54]. However, the observations that SCE but not GC is the most frequent HR occurring during replication stress suggest that SCE may be more critical for repairing PARP1 inhibition associated lesions. In addition, the results from Schultz et al. suggest that GC following induction of a site-specific DSB is normal in PARP1-inhibited cells. In contrast, PARP1 inhibition leads to an increase in crossovers as measured by SCE frequency in culture [127]. Cells isolated from PARP-1 knockout mice exhibit a hyper recombination phenotype and genetic instability in the form of increased levels of SCE, micronuclei and tetraploidy [128,129]. Moreover, the study from Bunting et al. also demonstrated that PARP inhibition caused an increased SCE in MEF cells [84]. All of these studies suggest that SCE is involved in repairing DNA lesions caused by PARP inhibition. BRCA1 promotes SCE-mediated HR in response to replication stress, which could be a mechanism explaining why BRCA1 deficient cells is hypersensitive to PARP inhibitors. Therefore, it is most likely that without PARP1, SSBs accumulate and then collapse replication forks to initiate SCE-mediated HR. If a PARP1 deficient cell is also deficient in BRCA1, SCE-mediated HR cannot occur, and the cell then dies or undergoes error-prone NHEJ (Figure 3). However, the possibility that the role of BRCA1 in GC is important for cell killing induced by PARP inhibitors cannot be excluded. In addition, it is also important to recognize that PARP activity and other proteins are also important for HR at stalled forks since it has been recently reported that PARP1 collaborates with MRE11 to promote replication fork restart, most likely by recruiting MRE11 to the replication fork to promote resection of DNA. Both PARP1 and PARP2 are required for HU-induced HR and cell survival after replication blocks [130]. 

## PARP1 resistance

Although PARP inhibitors displayed promising results for killing cancer cells with BRCA1/BRCA2 deficiency, there are several issues regarding PARP inhibitor-related therapies. Like other chemotherapy, acquired resistance to PARP inhibitors has been reported. The resistance to PARP inhibitors has led to the failure of phase III clinical trials in triple negative breast cancers[131]. Thus, there is urgency for elucidating the mechanisms by which resistance occurs. The acquired resistance to PARP inhibitors may be due to various mechanisms, including reverting inherited mutations in BRCA2 [132,133], an up-regulation of the *Abcb1a/b* gene encoding a P-glycoprotein efflux pump [134], and loss of 53BP1 which leads to restoration of impaired ssDNA resection resulting from BRCA1 deficiency [84]. In support of the idea that SCE-associated HR is required for repairing the DNA lesions caused by PARP inhibitors, 53BP1 depletion restores the decreased frequency of SCE because of BRCA1 deficiency [84]. How to overcome the acquired resistance to PARP inhibitors is a new direction for future study; strategies to overcome acquired resistance to PARP inhibitors has been discussed in review [124]. For instance, it has been reported that 6-thioguanine selectively kills BRCA2-defective tumors and overcomes PARP inhibitor resistance [135].

A second issue regarding PARP inhibitor associated therapy is that even if there is an observed sensitivity to PARP inhibitors in cancer cells without functional BRCA1, the difference is much smaller compared with the difference reported previously on pre-cancerous cells [136]. In addition, not all breast cancer patients with BRCA1 mutations respond to PARP inhibitors [137] and a substantial fraction of advanced BRCA1-mutant cancers are resistant to these agents. Therefore, the potential factor(s) contributing the effectiveness of PARP inhibitors in the cytotoxicity of breast cancer cells with mutant BRCA1 needs to be explored. Since SCE mediated HR appears to be important to repair PARP inhibitor-induced replication lesions, any factors which potentially regulate SCE should have an effect on PARP inhibitor-associated cancer treatment. The observation that the role of BRCA1 in promotion of SCE following replication fork collapse is more profound in cells depleted of ATR provides the possibility of sensitizing cancer cells without functional of BRCA1 to PARP inhibitors by ATR inhibitors. Thus, the status of cell cycle checkpoints should be taken into account when PARP inhibitors are applied.

## Conclusions and perspectives

Mitotic HR promotes genome stability through the precise repair of DNA DSBs and other lesions that are encountered during normal cellular DNA replication and replication stress. Deficiency in HR provides a promising target for cancer therapy. It has become apparent that HR repair produced by replication arrest is different to that required for repairing classical two-ended DSBs. In the past, research has been focused on the role of BRCA1 in classical two ended DNA DSB repair by HR. Recent studies suggest that BRCA1 is critical for several subtype HR pathways following replication arrest. However, how BRCA1 acts in HR when replication forks stall/collapse has not yet been satisfactorily answered. Hence, further studies are needed to focus on the regulatory mechanisms of HR repair by BRCA1 in response to DNA replication stress in different settings. Any advance regarding this topic will benefit our understanding of the mechanisms underlying BRCA1 associated tumorigenesis, as well as the development of therapeutic approaches for cancer patients with dysfunctional BRCA1.

## Abbreviations

PARP: Poly(ADP-ribose) polymerase; DSBs: DNA double strand breaks; DSBR: Double-strand break repair; SDSA: Synthesis-dependent strand annealing; IR: Ionizing radiation; BER: Base excision repair; NER: Nucleic acid excision repair; HR: Homologous recombination; SSA: Single strand annealing; MMR: Mismatch repair; NHEJ: Non-homologous end joining; ssDNA: Single stranded DNA; HJ: Holliday junction; GC: Gene conversion; SCE: Sister chromatid exchange; IRIF: IR induced focus; MMC: Mitomycin C; HU: Hydroxyurea; TLS: Translesion DNA synthesis; aCGH: Array-comparative genomic hybridization; MEF: Mouse embryonic fibroblast cells; SKY: Spectral karyotyping.

## Competing interests

The author declares no competing financial interests.

## Authors’ contributions

JZ drafted the manuscript and approved the final manuscript.

## Authors’ information

JZ is currently an assistant professor in the Department of Radiation Oncology, Case Western Reserve University School of Medicine. JZ has more than ten years of experience in homologous recombination.
